# Supplementary figures and images for: Nitrogen coordinated import and export of arginine across the yeast vacuolar membrane
Source: PLoS Genet. 2020 Aug 10;16(8):e1008966. doi: 10.1371/journal.pgen.1008966 (PMC7440668; doi:10.1371/journal.pgen.1008966)

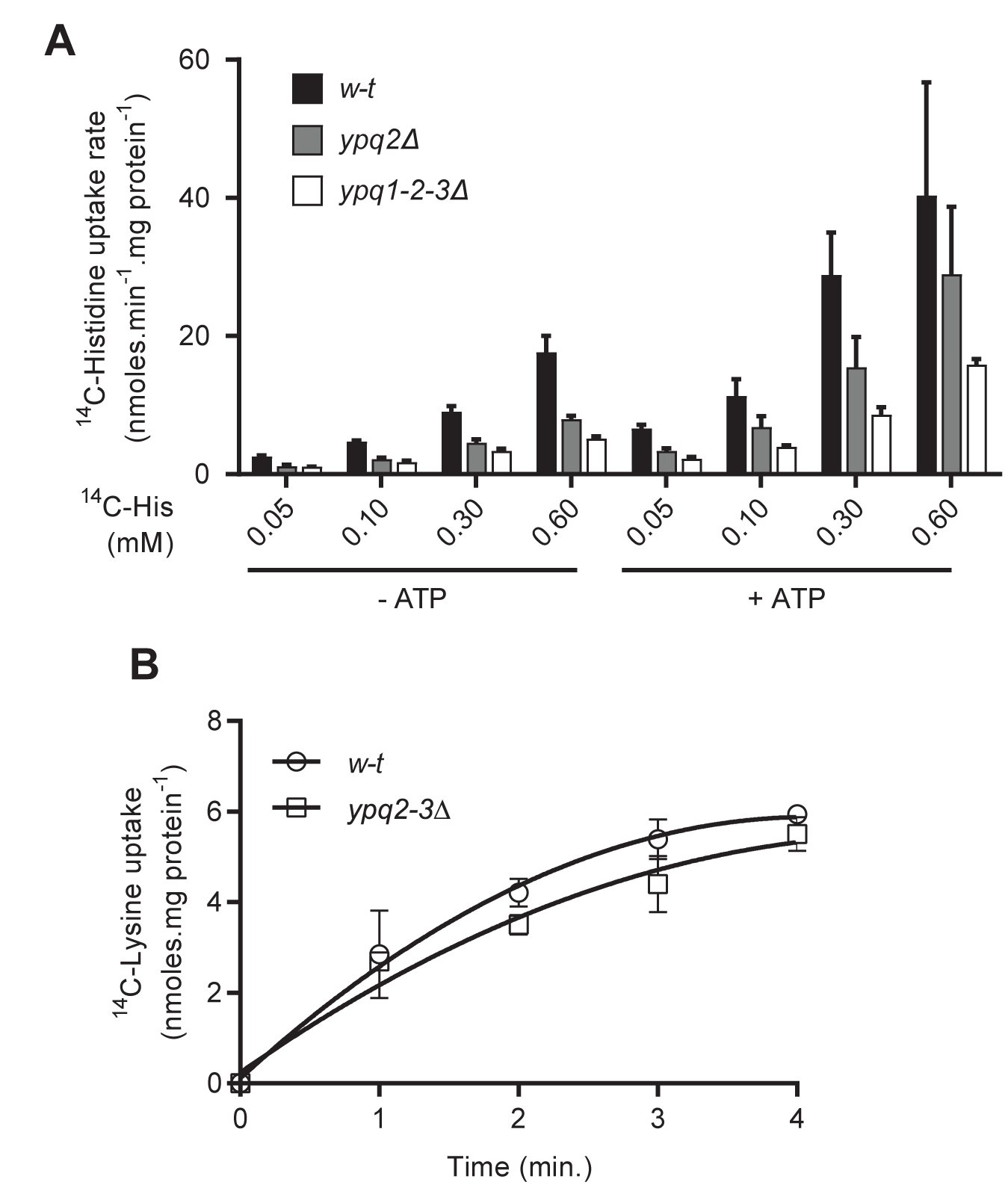

Supplement: S1 Fig — (A) The accumulation, in vacuoles isolated from w-t, ypq2Δ and ypq1-2-3Δ strains, of 14C-L-His added at different concentrations (mM) was measured after a 4-minute incubation. The vacuoles were incubated for 8 minutes in the absence or presence of ATP (4 mM) before addition of 14C-His (n = 2). (B) Time course of 14C-Lys (100 μM) uptake into intact vacuoles from the w-t and ypq2-3Δ strains in the absence of ATP. (TIF) [file pgen.1008966.s001.tif]

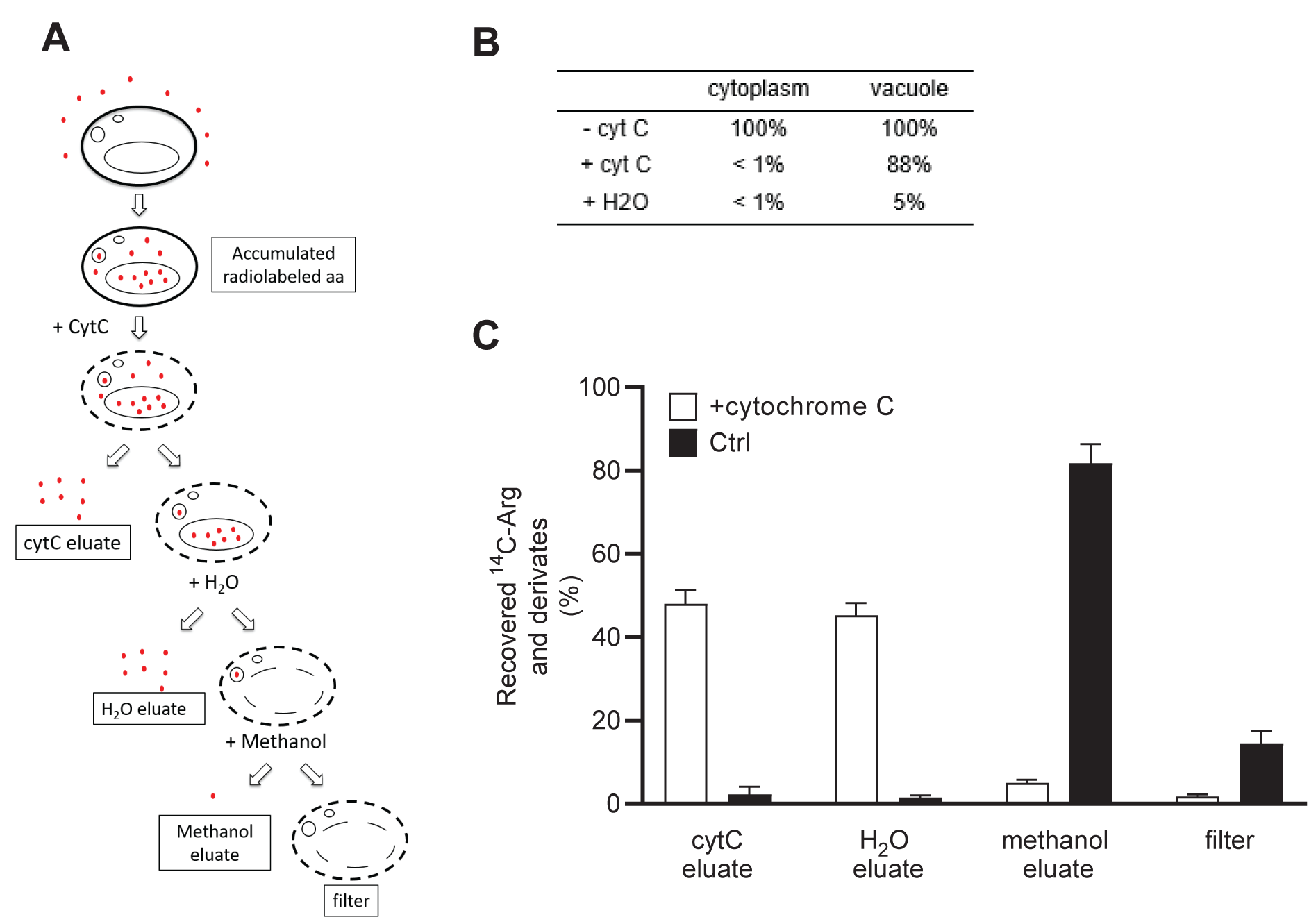

Supplement: S2 Fig — (A) Schematic of the permeabilization assay protocol. After uptake of the radiolabeled compound, cells were permeabilized with cytochrome C and filtered. This allowed extraction of soluble amino acids and other metabolites present in the cytosol (cytC eluate). A subsequent osmotic shock with H2O led to permeabilization of the vacuolar membrane and release of the vacuolar content (H2O eluate). Any remaining intact cell membranes were then disrupted with 50% methanol (Methanol eluate). Lastly, the radioactivity contained in all eluates and the filter was measured. (B) Percentages of cells showing a CMAC-labeled cytoplasm or vacuole under control conditions (-cytC), after cytochrome C permeabilization of the plasma membrane (+cytC), and after permeabilization of the vacuole (+H2O). (C) Fractions of the 14C-Arg and derivatives recovered during the permeabilization procedure. After uptake of 14C-Arg (38 μM), w-t cells underwent the full permeabilization procedure with either the permeabilization buffer containing cytochrome C or a control buffer with no cytochrome C. In the absence of cytochrome C in the buffer, most of the internalized radiolabeled compound was recovered in the Methanol eluate, suggesting that the cells were not permeabilized and that methanol effectively disrupted the cell membranes. In the presence of cytochrome C, most of the radioactivity was recovered in the cytC and H2O eluates and only a small fraction was eluted when methanol was added. (TIF) [file pgen.1008966.s002.tif]
