# Supplementary material for: Nitrogen coordinated import and export of arginine across the yeast vacuolar membrane
Source: PLoS Genet. 2020 Aug 10;16(8):e1008966. doi: 10.1371/journal.pgen.1008966 (PMC7440668; doi:10.1371/journal.pgen.1008966)
Supplement: Attachment — Submitted filename: Cools et al - p by p to reviewers final.docx [file pgen.1008966.s003.docx]

Nitrogen coordinated import and export of arginine across the yeast vacuolar membrane

Melody Cools, Simon Lissoir, Elisabeth Bodo, Judith Ulloa-Calzonzin, Alexander DeLuna, Isabelle Georis, Bruno André

**Point-by-point description of how we addressed the remarks of reviewers #1 and #2**

Reviewer #1 (Comments to the Authors):
Reviewer #1: This is a description of a well carried out study of how vacuoles help cells store and mobilize organic nitrogen. Two important gene-function relationships are identified, and kinetics and/or other important characteristics of their encoded transporters are described. Furthermore, the authors show the nitrogen status to have strong regulatory effects on the two transporters in a way that makes biological sense, although the mechanisms are yet obscure.
The manuscript is also well-written, creating a flow where generally the results from one experiment suggest questions that are addressed in the next one. This makes it easy and pleasant to read.

We thank Reviewer #1 for these positive comments, and also for the provided in-depth analysis of our manuscript.

I have one general comment. The exchange function of Ypq2 is pointed out in a slightly wrong way. At places, the reader will think that Ypq2 has two states, one in which it is a uniporter and another where it exchanges molecules; that is in my view unlikely and would be a complicated assumption. All observations agree with the highly efficient exchange reaction being a side reaction that will always take place when there is Arg on both sides. It requires no energy, and there is no reason to think that it has any important biological consequence. Such exchange is also named counterflow and is a priori expected to be very commonly occurring. Let us assume that the function important for fitness in nature is uniport (export in the case of Ypq2) function and not exchange. All you need to explain the occurrence of a much higher rate of exchange than rate of net transport is a kinetic bottleneck in the conformational shift of the transporter without ligand between the inward-open conformation and the outward-open conformation. Such bottlenecks should a priori be expected to commonly arise in evolution on a seemingly random basis in various steps of transport cycles including the conformational shift mentioned, and indeed counterflow is often found in transporters when it is looked for, also when it has no apparent biological significance. Not only uniporters exhibit counterflow; good old LacY also has it (see e.g. Guan L, Kaback HR (2006) Lessons from lactose permease. Annu Rev Biophys Biomol Struct 35:67–91). I encourage you to state this or parts of it, perhaps in the Discussion. I have below indicated a few places in the manuscript where changes should be made with this view; there may be others. Nevertheless, I fully agree that the exchange function is an important part of the story that contributes to the flow guiding the reader.

We thank the reviewer for this relevant comment and we agree that the proposed description of Ypq2’s transport mechanism is more suited. We thus modified two parts in the manuscript to reflect how the Ypq2-dependent Arg/Arg exchange measured in our in vitro assays reflects a side reaction that stems from Arg being present on both sides of the membrane rather than a switch from one state to another. See lines 204-210 and 535-542.

I have many minor comments:
P1 Italicize “VSB1” *-> done*
Line 4 Consider changing “, and” into a semicolon. *-> instead of a semicolon, we propose to split the original sentence in two (lines 3-4).*
15 Insert a comma after “nutrients” and another one after “arginine”, in both cases to indicate a non-defining relation. *-> done (line 16)*
76 Change “VTC” into “vacuolar transporter chaperone (VTC)”. *-> done (line 77)*
77 Change “mM)” into “mM phosphate groups)”. *-> done (lines 77-78)*
103 Consider changing “seemed” into “appeared”. *-> done (line 104)*
125 I suggest changing “are” into “appear”, since the experiments described immediately above concern a single amino acid only. *-> done (line 124)*
136-140 First, you do not at this point of the story need to indicate one interpretation as being simpler than others. Second, if you wish to list possible explanations here, you need to mention a third, and in my mind even simpler, possible explanation, namely the one you reach later in the manuscript: [14C]Arg uptake can take place through Ypq2 by exchange with intravacuolar amino acid(s). *-> we agree with this comment and we thus deleted this statement*
149 Change “w-t” into “w-t (23344c)” here and perhaps other places. -> we think that this would render the text unnecessarily cumbersome. To take into account this comment, we have emphasized in Materials and methods that “The reference wild-type (23344c) and the mutant strains used in this study (Table 2) derive from the wild-type strain ∑1278b [47]”.
171 Change “Unsurprisingly, cold” into “As expected, non-radioactive” in order to make text understandable to readers unfamiliar to the convention “hot”-“cold”.

- > done (line 165)

… Furthermore, change “neutral amino acids such as” into “the neutral amino acids”. I agree that it is likely that other neutral amino acids behave the same, but you should not conclude it unless of course you tried them, in which case you should describe it -> we agree with this point. The sentence now states “.. , and two tested neutral amino acids (isoleucine and alanine) did not significantly inhibit this uptake” (lines 165-166).
180 and 205 Insert “primarily” or “mainly” after “is”. - > done (lines 174 and 199)
191 Change “previously described,” into “described for intact vacuoles,”. -> done (line 185)
199 Change “not.” into “not, although ATP stimulated the uptake.”. -> done (line 192)
204 Insert “into vacuole-derived vesicles” after “uptake”. - > done (line 198)
210-212 **This is an overstatement.** Instead, it would be correct to say “Appreciable Ypq2 activity does require, however, the presence of at least one of its substrates inside the vacuole, since little 14C-Arg was taken up by vacuolar vesicles devoid of Arg or His (Fig 3C).”, of course with “14” in superscript. - > we agree with this comment and have modified this statement (lines 204-207)
213-215 **This is also an overstatement**; it gives a wrong impression of the content of the three references given. None of the two first references mention a switch. In each case, a mitochondrial carrier is described which has a high kcat for exchange and a much lower yet significant kcat for uniporter activity. It is expected, and not a consequence of a shift of mechanism, that the relative significance of the latter becomes larger when the concentration ratio across the membrane increases. The third reference, on GLUT1, does describe a switch, but also in this case is the statement misleading. The effect of lowering the external glucose concentration has a large delay and occurs indirectly via the ATP/AMP energy system, a fact that in my view makes this reference less relevant; there is nothing that points to a similar mechanism for Ypq2. Most unprepared readers who do not read the three cited papers will erroneously think that the papers show cases where a high concentration ratio of a ligand (transport substrate) directly causes a change of transporter mechanism. This is not so; in references 30 and 31 one just sees a consequence of different experiments emphasizing different capabilities of the protein in question. The wording must be corrected, see my general comment and e.g. my comment to line 977. - > we thank the reviewer for drawing our attention to this important point. This part of the text was revised and the three references were discarded (lines 206-209)
223-225 Modify wording. I do not see that the hypothetical pH profile itself argues for the model, but it is indeed important to point out as an explanation for the effect of ATP, based on evolutionary considerations. I agree that, otherwise, readers might at this point think that the effect of ATP suggests that the pmf can drive transport through Ypq2, which I also agree appears unlikely -> We agree with this comment and have modified this statement (lines 215-218)
261 Insert “than” after “more” -> done (line 240)
297 I encourage you to reserve the name with SGD. -> We thank the reviewer for this suggestion. This has now been done.
457 Consider changing “our observations show” into “we find”; the first could to some readers mean all observations described in the manuscript. -> we changed into “these observations” (line 451)
461 Consider changing “is” into “shows up as”. -> done (line 455)
533 Make “4” subscript.-> this was deleted in order to reduce the length of the discussion
595 “adopt” is misleading, see my general comment, and reconsider reference 32, see my comment to lines 213-215. -> this part of discussion has been changed accordingly, taking into account the general comment (lines 535-542)
600 Also here, please rephrase slightly and avoid “switch” -> done (lines 539-540)
611 Insert “the” before “protein”.-> done (line 551)
657-659 The nomenclature for PS buffer must be clarified. Strictly interpreted, the layer e.g. immediately over the lysate should contain 15+8 percent Ficoll, but I think it only contains 8. -> done (lines 587-589)
665 Change “O” to lower case, italic, since it means ortho; otherwise some readers will think it refers to binding to an oxygen atom. Another common name that you can choose is 1,10-phenanthroline. You can also add a vendor or producer and a catalog number -> done (changed into 1,10-phenanthroline (Merck; catalogue number: 131377) (lines 593-594).
669 and 996 Insert comma before “and”, since a new independent sentence starts. -> done (lines 598 and 944)
691 Consider changing “Corrected for” into “normalized to”. -> done (line 620)
704-705 Specify the scintillation liquid. Counting efficiency depends on the identity of the scintillation liquid, the sample solvent, and the mixing ratio. -> done (line 633)
736 Change “m” into “nm”. -> done (line 676)
804 Delete “ (80-)”.-> done
819 Change “enzymology” into “Enzymology”, and consider changing “177-96 p” into “pp 177-96”. Check full reference list for similar cases. -> done
821 Change “Vacuolar” into “vacuolar” and “Cellular” into “cellular”. Check full reference list for similar cases. -> done
831 Make the “+” superscript. Check full reference list for other cases of possible subscripts and superscripts. -> Reference was deleted
955 Change “Arg uptake into” into “Arg uptake through Ypq2 into isolated”. -> done (line 902)
955-957 Specify in this experiment whether ATP was present or absent. -> done (lines 903-904)
956-957 and 959 Change “ (see text for the strains used)” into “. Values for the ypq1-2-3Δ strain were subtracted from those for the ypq1-3Δ strain”, of course with the genotypes in italics. -> done (lines 904-905)
958 Insert “of Ypq2” after “selectivity”. -> done (line 906)
963 Change “Vacuoles were” into “Vesicles were”. -> done (line 911)
965, 1049, 1052 and 1069 and Table 1 header: Make “14” superscript. -> done
967, 971, 1007, 1010 and 1046 Change “ctrl” into “Ctrl”. -> done

967, 971, 1008 and 1011 Consider changing “cold” into “non-radioactive”. -> changed to “non-radiolabeled”
977 Change “Ypq2 switches into an exchange mode of transport” into “exchange will dominate, and net transport will slowly cease”. -> this figure panel was deleted
1025 Change “w-t and vsb1Δ” into “w-t (SL073) and vsb1Δ (SL074)”. -> done (line 974)
1032 Change “ctrl” into “control (Ctrl)” or “untreated (Ctrl)”. -> done (line 981)
1085 Insert space between number and unit. Check full manuscript for similar cases. -> done (line 1032)
Figures: In micrograph panels, either insert an indicated 10-micrometer bar (one per figure is sufficient) or indicate the length of sides of the panels. -> done
Fig. 1A: Explain in the legend the scissors depicted in right panel. -> done (line 878-879)
Fig. 4A: **Please delete this.** The text is easy to understand by itself and can even be improved, and the two drawings make more confusion than explanation. There are at least two problems with them. First, the exchange reaction has no consequence on any concentrations; it is the uniporter function for both directions that assures that an equilibrium is approached. Second, the membrane potential and the negative charges of the polyphosphates make it unlikely that a cation like Arg will have the same concentration on both sides at equilibrium. -> We agree with the reviewer and the figure was deleted
Fig. 4C: The ordinate axis of the right panel needs some explanation for non-experts on survival curves to understand. Move, e.g., the explanation on s from Materials and methods to the figure legend. -> done (line 930-932)
Fig.5A: Most genotype designations here end correctly with a delta, and that was intended also under the rightmost open bar. However, on my screen the character is erroneously a framed cross. -> done
Fig. 5D: This is the first time the authors present CMAC. A brief explanation, at least of the purpose and principle, is needed in the legend. -> done (line 945)

Reviewer #2 (Comments to the authors):
1. The authors claim that Ypq2 is a transporter (Lines 177-179); however, neither they nor others have shown direct activity of this protein, they probe activity in system (intact vacuoles) containing all or most of vacuolar protein. It would be important to stress this in their manuscript.

We agree with the reviewer that to provide absolute proof that Ypq2 is a transporter, experiments with the purified protein reconstituted in vesicles are needed. This is now commented in the discussion (lines 522-524). However, several independent studies concerning Ypq2 and homologs from other species are in line with a role of these proteins as Arg transporters (see lines 514-522 in Discussion). For these reasons, we think that it is reasonable to present Ypq2 as a membrane transport protein.

2. Along the same line, the authors show that Ypq2-dependent uptake is dependent on the intra-vacuolar concentration of arginine (Figure 3B and C) and propose that transport is bidirectional, and only unidirectional during starvation, although this was not assessed. Interestingly, they are able to detect activity even in the control condition that, although it is low, it is still stimulated by ATP. Can the authors rule out that the proposed exchange reaction is carried out by more than one transporter and that Ypq2 is rather the importer?

We agree with the reviewer’s suggestion that Ypq2 is not the sole protein involved in Arg transport across the vacuolar membrane. This is evidenced by the residual Arg uptake measured in intact vacuoles of the quadruple ypq1-3Δ vsb1Δ mutant when the amino acid is used at high concentrations (Fig. 6A), and the residual export of Arg detected in vacuole-derived vesicles filled with Arg and isolated from the ypq2∆ mutant (Fig. 4B, added as suggested in point 3). This has now been well emphasized in the text (lines 144-150, 510-513).

Regarding the possibility that Ypq2 could only function as an importer, we present several clues arguing the opposite: (i) intracellular Arg levels in the ypq2Δ mutant are higher than in the w-t strain (Fig. 5A), a result more compatible with a role of Ypq2 in Arg export (ii) Ypq2 plays an important role for the utilization of intravacuolar Arg stocks under N limiting conditions (Fig. 4A) (iii) Ypq2 is not necessary to Arg accumulation in the vacuole under steady state conditions (Fig 8C) (iv) we measured the export of radiolabeled Arg trapped in vacuole-derived vesicles and found that Ypq2 importantly contribute to this activity (Fig. 4B, added as suggested in point 3). Taken together, the results allow us to rule out the possibility that Ypq2’s role is limited to Arg import. The transport properties of Ypq2 are commented in Discussion (lines 503-552).

3. The authors propose that Ypq2 would mobilize Arg from the vacuole to the cytosol during nitrogen starvation (Figure 4). They convincingly show that Ypq2 is required for intracellular consumption of arginine during starvation; however, they do not show direct Ypq2-dependet export from the vacuole. To address this a bit more directly, the authors could measure the efflux of radioactive arginine that was previously incorporated into vacuoles before to demonstrate Ypq2-dependent export of this amino acid. Additionally, the export could be assessed under conditions of nutrient rich and starvation conditions.

*We thank the reviewer for this suggestion. As just mentioned above, we addressed this point by measuring the export rate of Arg (50 µM) incorporated into vacuole-derived vesicles prepared from the w-t and ypq2*Δ *mutant strains, and found that it is ~2-fold reduced in vesicles from the ypq2*Δ *strain. This is illustrated in Fig. 4B and commented in lines 241-252. The vesicles used in these experiments were prepared from cells grown under nitrogen rich conditions. We don’t think that it is useful to repeat the experiment using vesicles from nitrogen-starved cells because Ypq2 is shown to be active under these conditions.*

4. The assumption that Ypq proteins are arginine transporters seem to be supported by the fact they homologues to the PLQ2 mammalian transporter. For PLQ2 it has been shown that mutation in P55 abolished arginine uptake. To demonstrate homologous activity, the authors could mutate the equivalent position in Ypq2 to test if the mutant is able or not to rescue the uptake phenotype they observe.

*We thank the reviewer for this suggestion. This Ypq2(P29A) mutant, equivalent to the above-mentioned PQLC2 mutant, has in fact been isolated and described in a recently published study (Kawano-Kawada et al. 2019). As expected, this Ypq2 mutant lost its transport function. We have now included this information in Discussion, in a paragraph where the role of Ypq2 as a transport is commented, as recommended (lines 514-522).*

5. The authors claim that Vsb1 is a transporter, but do not show transporter activity. Although I do not expect them to demonstrate activity in vitro, I find it important that they remain cautious with their interpretation as also in their statements along the manuscript, as they refer to this protein as an essential transporter.

We took into account this issue by avoiding such statement throughout the text, including in the abstract (e.g. lines 5-6, 24, 86-87, 291-308, 319, 406-408, 468…). Additionally, we state more clearly in Discussion that additional experiments are needed to confirm that Vsb1 indeed catalyzes a transport reaction (lines 482-486), although we now also mention that Vsb1 does belong to a well-documented superfamily of transporter (lines 5-6, 291-292).

6. Figure 7: The analysis using bafilomicin A was performed in wt cells, so it is difficult to claim that the block in V-ATPase function specifically affects the activity of Vsb1. Please rephrase.

*The interesting result provided by this experiment is that wt cells treated with bafilomycin A (Fig. 7G) displayed a phenotype very close to that of untreated vsb1 mutant cells (Fig. 7E). We have now modified the text to emphasize this point. To more directly address the point raised by the reviewer, the manuscript has also been modified (lines 397, 403, 476-477) to reflect a more cautious view from this observation.*

7. Along their entire manuscript the authors mix up a few things in my view. They observe under different conditions (N-depleted/repleted) different requirements of their transporters, and do so by comparing deletion mutants with wild-type. They then conclude that each of the transporters is regulated (in abstract and throughout the manuscript). However, they have no evidence at all that this is the case. Results from Figure 8 do not strictly show that these proteins are inactive in each condition, they are just not required. I agree that nitrogen regulation may be likely, but they do not show regulation of either transporter, and they should thus rephrase these passages accordingly.

To take into account this issue, we have modified the text to reflect a more nuanced view (including in Abstract) and to emphasize that more evidence is needed to confirm that a regulation of Vsb1 and Ypq2 occur according to N availability. See lines 8, 12, 88, 405, 424-425, 452-453, 488-489, 553-556.

8. I found the entire discussion too long and too excessive. The repeat many aspects of their results section. I recommend to condense it strongly (by ½) to make to more accessible.

We agree with the reviewer that the discussion is quite long and have reduced it by about 1/3. Since we think our study leaves much to be discussed and since PLOS genetics articles don’t seem to have a limit to the length of the discussion, we propose to not reduce it further.

Minor issues 1-3.

1. Line 124: “…the intact yeast vacuoles isolated via the above methods…”. The authors refer to the “above methods” as if they would explain them, but they do not describe the isolation method itself, so it would be suitable to say “our method” or “the used method”.

2 .Paragraph between lines 112-115 can be interpreted as they could not establish the pH measurement when incubated with the inhibitor. “ the pH measured in isolated vacuoles became more acidic in the presence of ATP (Fig 1), this acidification was partially lost upon addition of the K+/H+ ionophore nigericin, and it could not be established if the vacuoles were pre-incubated with the V-ATPase inhibitor bafilomycin A (Fig 1C)”

3. Figure 7C: It would be helpful for readers if the authors name of the protein fused to GFP (Can1) also in the figure.

*We agree with these three comments and have made the appropriate modifications in the text.*

4. Many experiments have been repeated only twice. Particularly, Figures 3B and 6D show the same control condition but the effect of ATP is only evidenced in Fig 3B. In this case as in other in which the n=2, at least 3 repeats should be included.

We fully agree with the reviewer that the data of at least three biological replicates are in principle needed. This is already the case for more than half of our experiments. However, we could not, for this study, strictly follow this rule for all experiments, and we would like to explain why. Most of these experiments require painstaking preparation of vacuoles from large volumes of culture. Although we take suitable precautions to prepare yeast cultures and apply our vacuole prep protocol strictly under the same conditions, we regularly encounter the problem that extraction yields are insufficient to carry out transport assays. That is why, when two independent biological replicates gave coherent data and only limited variations, a third replicate has sometimes not been considered. That being said, we wish to stress that for several experiments where n=2, a third experiment has been carried out for some of the tested conditions. For instance, in Figure 2C, the data of the “w-t” correspond to those of three independent biological replicates. Additionally, for the remaining experiments where n=2, preliminary assays gave data fully consistent with those of the two final experiments carried out to prepare the figures of the article.

To take into account this issue raised by the reviewer, and for the sake of transparency, we have now included a note “Reproducibility of experiments and statistics” in the Materials and methods to clarify that some experiments have been limited to 2 biological replicates. We thus hope that the reviewer, who included this request in the list of “minor issues”, will accept to consider our manuscript in its current version.
